# The potential therapeutic roles of Rho GTPases in substance dependence

**DOI:** 10.3389/fnmol.2023.1125277

**Published:** 2023-03-30

**Authors:** Qin Ru, Yu Wang, Enyuan Zhou, Lin Chen, Yuxiang Wu

**Affiliations:** Department of Health and Physical Education, Jianghan University, Wuhan, China

**Keywords:** Rho GTPases, substance dependence, GTPase-activating proteins, guanine nucleotide exchange factors, cytoskeleton remodeling

## Abstract

Rho GTPases family are considered to be molecular switches that regulate various cellular processes, including cytoskeleton remodeling, cell polarity, synaptic development and maintenance. Accumulating evidence shows that Rho GTPases are involved in neuronal development and brain diseases, including substance dependence. However, the functions of Rho GTPases in substance dependence are divergent and cerebral nuclei-dependent. Thereby, comprehensive integration of their roles and correlated mechanisms are urgently needed. In this review, the molecular functions and regulatory mechanisms of Rho GTPases and their regulators such as GTPase-activating proteins (GAPs) and guanine nucleotide exchange factors (GEFs) in substance dependence have been reviewed, and this is of great significance for understanding their spatiotemporal roles in addictions induced by different addictive substances and in different stages of substance dependence.

## 1. Introduction

**Figure 1:**
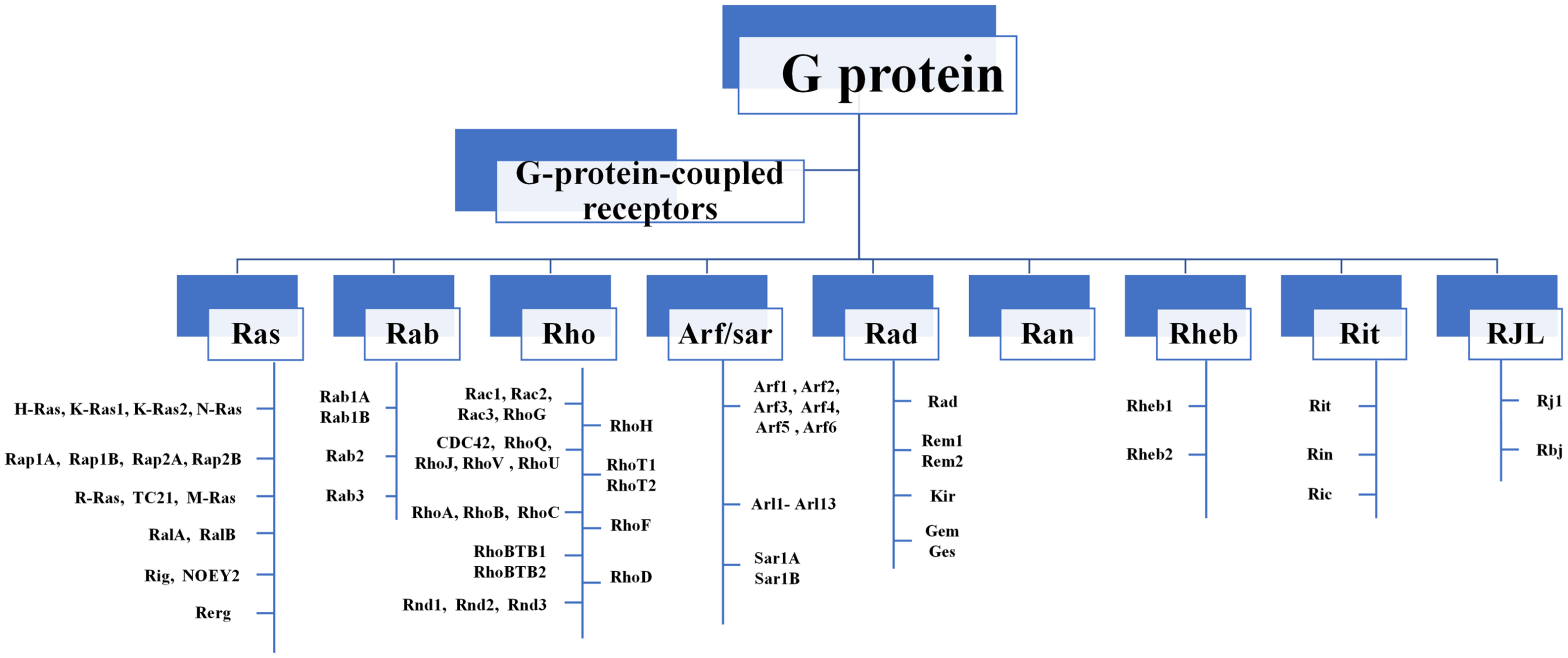
The composition of Ras-like superfamily. There are nine major subgroups in the Ras-like superfamily, including Ras, Rab, and Rho families. Rho GTPases can be divided into 9 subfamilies (Rac, Rho, RhoD, Cdc42, RhoH, RhoF, Rnd, Miro, RhoBTB) according to their structural and functional differences.

The Ras-like superfamily G proteins are monomer proteins of approximately 21 kDa that function as molecular switches to regulate a wide range of primary and versatile cellular processes (Shutes and Der, 2004; Gao et al., 2019; Casalou et al., 2020). There are nine major subgroups in the Ras-like superfamily, including Ras, Rab, and Rho families (Figure 1). Even between distantly related clades, members in the Ras-like superfamily share 65–85% amino-acid sequence similarity (Rojas et al., 2012). This extreme conservation is inconsistent with their roles in fundamental cellular functions such as F-actin dynamics and endo/exocytosis. The Ras-like proteins rely on their structural changes between guanosine triphosphate (GTP)-binding conformation and guanosine diphosphate (GDP)-binding conformation, acting as binary signaling switches biochemically, and transmitting upstream signals to downstream molecules (Bosco et al., 2009; Raimondi et al., 2011).

**Figure 2:**
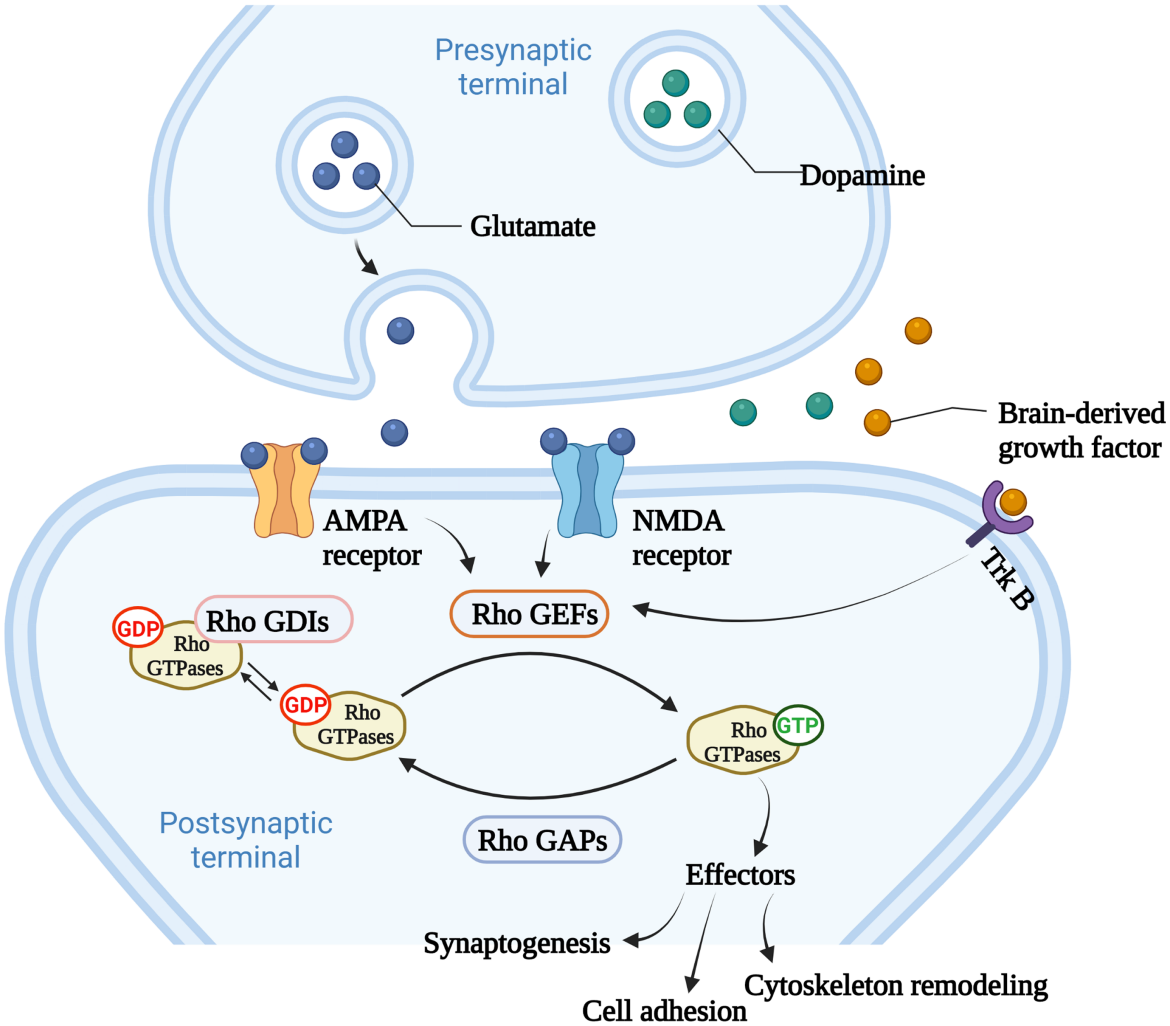
The main function of Rho GTPases. Rho GTPases cycles between an active GTP-bound state in the cell membrane and an inactive GDP-bound state in the cytoplasm, and the activity of Rho GTPases is regulated by Rho GTPases guanine nucleotide exchange factors (Rho GEFs), Rho GTPase activating proteins (Rho GAPs), and Rho GTPases guanine nucleotide dissociation inhibitors (Rho GDIs). Rho GEFs catalyze the conformational change of Rho GTPases and activate Rho GTPases. Rho GAPs facilitate the hydrolysis of GTP into GDP within Rho GTPases, and deactivate Rho GTPases. Rho GDIs inhibit the dissociation of GDP from Rho GTPases and stabilize the inactivation of Rho GTPases. Rho GTPases act as important molecular switches to transmit signals to downstream effectors that regulates numerous cellular functions, such as cytoskeleton remodeling, cell adhesion and other essential life processes.

Among Ras-like superfamily, the Rho family (Rho GTPases) with 22 members are primary regulators of the cytoskeleton remodeling, cell adhesion, polarity, and locomotion processes (Vega and Ridley, 2007). Rho GTPases are divided into nine subfamilies (Rac, Rho, RhoD, Cdc42, RhoH, RhoF, Rnd, Miro, and RhoBTB) according to their structure and function differences (Vega and Ridley, 2007). Like other members of Ras superfamily, most Rho GTPases cycle between an active GTP-bound state in the cell membrane and an inactive GDP-bound state in the cytoplasm. Activated Rho GTPases can contact with a variety of downstream effectors that regulates numerous cellular functions, such as cytoskeleton remodeling, cell motility, cell polarity, vesicle transport, cell adhesion and other essential life processes (Jung et al., 2020; Figure 2). Therefore, they are considered to be molecular switches of receptor signaling pathways on the surface of synapses, and contribute significantly in regulating the synaptic structure and neuron development, and are closely related to cognitive and emotional disorders and neurodegenerative diseases.

The high relapse rate of substance addiction mainly lies in the fact that addiction will form lasting, stubborn and environment-related memories in the human brain. The continuous morphological plasticity of dendritic spines and the functional plasticity of excitatory synaptic transmission may be the neurobiological mechanism of the abnormal behaviors induced by addictive substances. Preliminary studies showed that Rho GTPases might participate in the acquisition and extinction of addictive substances-associated contextual memory (Tu et al., 2019; Zhao et al., 2019). Therefore, in this review, we focused on several extensively investigated Rho GTPases involved in substance addiction. By detecting expression levels of these Rho GTPases and potential protein-protein interactions, we attempted to explore how these Rho GTPases and their modulators regulate specific signaling pathways spatiotemporally to affect changes in neuron structure and functional plasticity, thus participating in the pathological process of substance addiction.

## 2. The regulation of Rho GTPases

The activities of Rho GTPases are mainly controlled by three classes of regulators. The first-class is Rho GTPases guanine nucleotide exchange factors (Rho GEFs). Rho GEFs are classified into two subclasses, including dedicator of cytokinesis (DOCK)-related proteins (Benson and Southgate, 2021) and Dbl-like proteins, whose structure has similarity to the Dbl (diffuse B-cell lymphoma) protein (Jaiswal et al., 2013). Most Rho GEFs share a Dbl homology (DH) domain (170–190 amino acids) and Pleckstrin homology (PH) domain (approximately 100 amino acid). DH domain is responsible for the guanine nucleotide exchange activity on Rho GTPases, and the binding of DH domain to GTPases promotes the combination of Rho GTPase to GTP and catalyzes its conformational change, which can then activate a set of downstream effectors for signal transduction (Jaiswal et al., 2013). The PH domain anchors GEFs to the membrane by interacting with specific lipids on the membrane, then induces orientational and/or conformational changes on the membrane surface and activates Rho GTPase by aligning the DH domain to a particular cytoskeletal position to mediate cell transformation (Zheng et al., 1996; Baumeister et al., 2003). Rho GTPase activating proteins (Rho GAPs), which are negative regulatory factors, promote the hydrolysis of GTP into GDP within the Rho GTPase and inactivate the Rho GTPase to terminate signal transduction (Amin et al., 2016). Rho GAPs have a conserved catalytic domain named Rho GAP domain (approximately 190 amino acids), which can supply a conserved “arginine finger.” Apart from conserved Rho GAP domains, Rho GAPs have several structural domains and sequence motifs, which take part in subcellular localization, lipid membrane association, and the connection to upstream signals (Jaiswal et al., 2014). Only a tiny amount of Rho GTPases are activated under normal physiological conditions, and the inactive Rho GTPases are associated with Rho GTPases guanine nucleotide dissociation inhibitors (Rho GDIs), which can isolate Rho GTPases in their GDP bound, inhibit the dissociation of GDP from Rho GTPase and stabilize the inactivation state of Rho GTPase (Matsuda et al., 2021). GDIs can be classified according to the specificity of G proteins, however, not all G proteins have GDIs (for example, the Rho and Rab families have GDIs, but the Ras family does not). Although different GDIs have some common structural and functional features, they usually have unrelated amino acid sequences, indicating that GDIs have appeared independently in the evolutionary process. To date, 3 Rho GDIs, 72 Rho GAPs, and 82 Rho GEFs have been identified in humans (Tcherkezian and Lamarche-Vane, 2007; Garcia-Mata et al., 2011; Amin et al., 2016; Fort and Blangy, 2017; Table 1). It is worth noting that post-translational modifications such as ubiquitination and phosphorylation can also regulate the activities of Rho GTPases (Abdrabou and Wang, 2018; Jung et al., 2020). These post-translational modifications and the regulators as mentioned above cooperatively determine the activation status, concentration, localization, and ability to bind downstream molecules of Rho GTPases.

**Table 1:** Addiction related Rho GTPase and their regulators.

Items	Number	Elements
Addiction related Rho GTPase	6	RHOD, RAC1, RAC2, RHOA, CDC42, RHOB
Addiction related Rho GEFs	13	FARP1, ARHGEF9, ARHGEF38, ALS2, ARHGEF12, ARHGEF2, PLEKHG2, ARHGEF3, ARHGEF1, ARHGEF6, AKAP13, SPATA13, RASGRF2
Addiction related Rho GAPs	7	ARHGAP39, OPHN1, BCR, ARHGAP28, SRGAP2, SRGAP3, ARHGAP6

## 3. The role of Rho GTPases on the regulation of synaptic plasticity

Synaptic connections between neurons are crucial to all aspects of neural activity and are therefore precisely regulated. Synaptic plasticity is the activity-dependent changes in the strength and efficacy of synaptic transmission across the synapses, and these changes are thought to underlie the neurobiology of addiction (Magee and Grienberger, 2020; Mateos-Aparicio and Rodriguez-Moreno, 2020).

**Figure 3:**
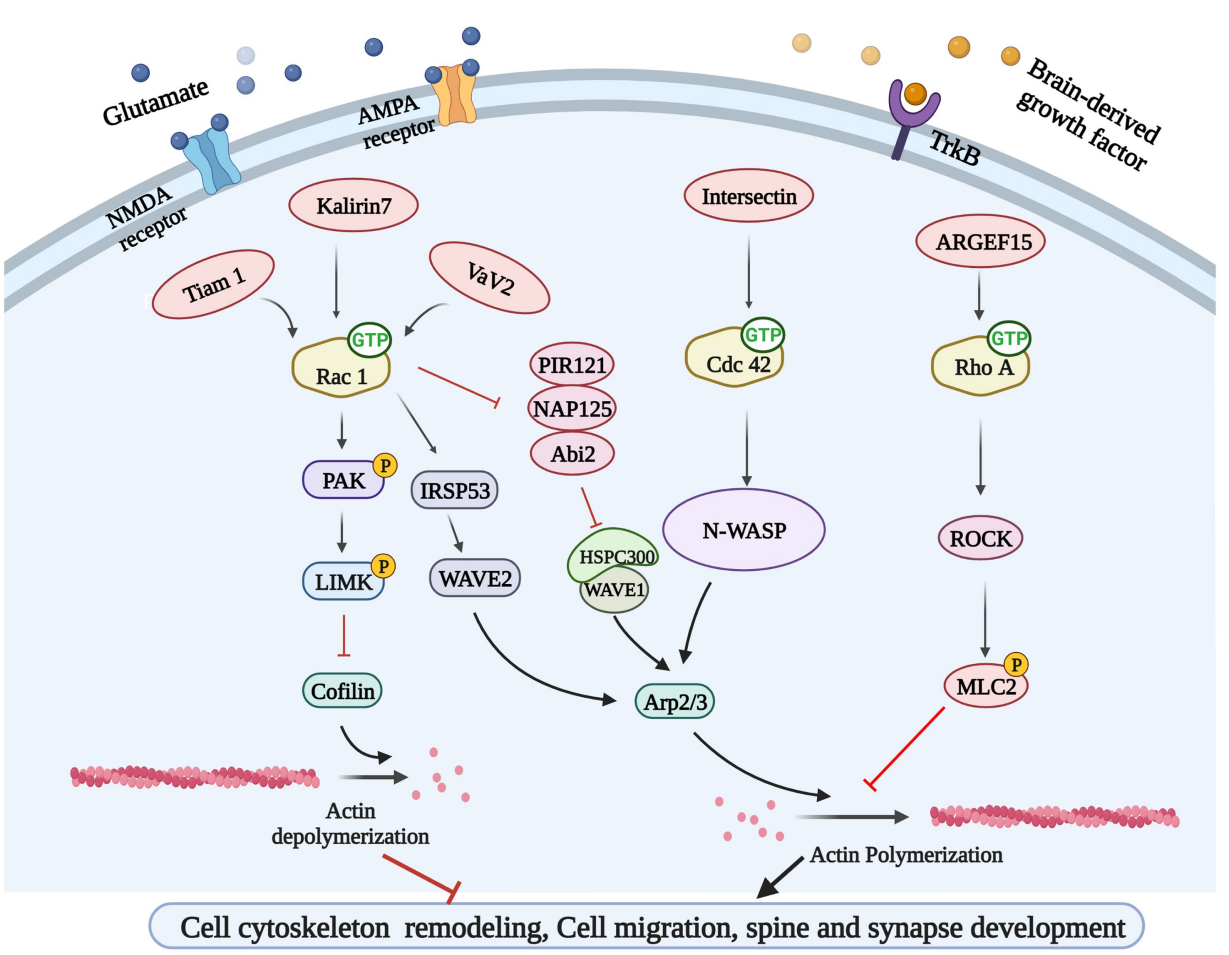
Rho GTPases-related signaling pathways. Glutamate and brain-derived neurotrophic factor (BDNF) could bind to their corresponding receptors and activate Rho GEFs, leading to Rho GTPases activation. Rho GEFs including Tiam 1, Kakirin 7, and Vav2 could activate Rac 1 and participate in cytoskeleton remodeling and synapse development through PAK/LIMK/Cofilin pathway and IRSP53/WAVE2 pathway. Rho GEF Intersection can activate N-WASP to regulate cytoskeleton remodeling by activating another GTPases Cdc42. ARGEF15 is also involved in this process by activating Rho A and its downstream ROCK.

The excitatory synapses of major neurons in the brain are mostly located at the tips of dendritic spines, which are dynamic structures, and the rapid remodeling of dendritic spines is essential for synaptic formation, function, and plasticity. Inappropriate dendritic spinous morphogenesis may lead to impaired information processing in the brain. Therefore, abnormal dendritic spines are associated with many neurodevelopmental, neuropsychiatric, and neurodegenerative diseases, and dendritic spine morphology can be a proxy for synaptic strength (Tolias et al., 2011). Dendritic spines are highly enriched in filamentous actin (F-actin), and the rapid remodeling of actin cytoskeleton determines the morphologic change ability of dendritic spines. Therefore, Rho-GTPases, which are well-known for their ability to control actin cytoskeleton dynamics, are undoubtedly an important part of the regulatory mechanism of dendritic spines morphogenesis (Duman et al., 2022). Ras-related C3 botulinum toxin substrate 1 (Rac1), cell division control protein 42 homolog (Cdc42) and Ras homolog family member A (RhoA) are the most studied members of the Rho GTPase family, and Rac1 and Cdc42 promote the formation, growth and maintenance of spine, while RhoA has opposite effects (Govek et al., 2005). For instance, cofilin cuts off F-actin, creating new barbed end for polymerization or causing actin depolymerization, which is necessary to regulate the density and morphology of mature spine (Cao et al., 2017). Serine/threonine kinase PAK1 is the main downstream effect of Rac1 and Cdc42. Rac1 and Cdc42 induce phosphorylation of LIMK and cofilin through PAK1 to inhibit cofilin activity, while inhibition of cofilin phosphorylation and F-actin polymerization resulted in loss of dendritic spines (Yang et al., 2018). The Arp2/3 complex is one of the most important intracellular actins nucleators, which forms actin networks and caps the ends of actin filaments (Chou and Wang, 2016), therefore, Arp2/3 is necessary for the formation and remodeling of actin cytoskeleton to regulate spine morphology and function. The function of the Arp2/3 complex is highly regulated by nucleation promoters such as Wiskott-Aldrich Syndrome protein (WASP) or WASP-family verprolin homologous protein 2 (WAVE2) (Stradal and Scita, 2006). Cdc42 activates Arp2/3 through N-WASP, which drives actin polymerization and dendritic spine development in neurons (Hasegawa et al., 2022). Similarly, Rac1 regulates Arp2/3 mediated actin polymerization by activating WAVE2 through IRSp53, and is involved in the formation and development of synapses (Ding et al., 2022). Rho-GTPases are essential for regulating excitatory synaptic formation and plasticity of mature synapses in neurodevelopment (Figure 3). Chronic substance dependence leads to behavioral, morphological and neurochemical plasticity changes that underlie compulsive drug-seeking behavior and relapse after withdrawal. Identifying and reversing the synaptic plasticity associated with addiction is considered a potential intervention strategy for drug addiction (Smith and Kenny, 2018; Speranza et al., 2021).

## 4. Rho GTPases and substance dependence

Abnormal morphology and density of dendritic spines could be observed in patients and animals with addiction (Beltran-Campos et al., 2015; Yin et al., 2020), accompanied by abnormal expression and activation level of Rho GTPase and its regulatory factors (Cahill et al., 2018). Regulation of Rho GTPase activities is very crucial for treating substance dependence (Table 2).

**Table 2:** Roles of Rho GTPases in substance dependence.

Substance	Behavior test	Strain	Nuclei or cell lines	Changes of Rho GTPases	References
Morphine	Conditioned place preference	C57BL/6 mice	Hippocampus	Number of dendritic spines ⇩ Rho A ⇧ ROCK⇧	Fakira et al. (2016)
	Withdrawal	C57BL/6J mice	Nucleus accumbens	Spines on medium-sized spiny neurons ⇩ Rho A ⇧	Cahill et al. (2018)
	Conditioned place preference and withdrawal	Sprague Dawley rats	Prefrontal cortex	Rac1 ⇧	Qu et al. (2020)
Heroin	Self-administration	Sprague Dawley rats	Dorsal hippocampus	RhoB⇧ ROCK⇧	Chen et al. (2017)
	Self-administration	Sprague Dawley rats	Dorsal hippocampus	Rac1 ⇧ F-actin/G-actin⇧	Chen et al. (2017)
	Behavioral sensitization	C57BL/6J mice	Nucleus accumbens	Rac1 ⇩	Zhu et al. (2016)
Cocaine	Goal-directed action	C57BL/6J mice	Prelimbic prefrontal cortex	Number of dendritic spines ⇩ ROCK⇧	Swanson et al. (2017)
	Locomotor activity	C57BL/6J mice	Prefrontal cortex	ROCK⇩	DePoy et al. (2013)
	Acute injection	CD-1 mice	Dorsal striatum, prefrontal cortex and hippocampus	Rnd3⇧	Marie-Claire et al. (2007)
	Locomotor sensitization	C57BL/6J mice × 129 mice	Nucleus accumbens	Rho-GEF Kalirin-7⇧	Kiraly et al. (2010) and Ma et al. (2012)
	Withdrawal	Sprague Dawley rats	Nucleus accumbens	Kalirin-7⇧ Rac-1⇧PAK⇧	Wang et al. (2013)
	Self-administration, locomotor activity and conditioned place preference	Mice	Nucleus accumbens	RhoGEF PDZ⇧ RhoA⇧ Rap1b⇧	Cahill et al. (2016)
	Behavioral sensitization and conditioned place preference	C57BL/10 mice	Nucleus accumbens	RhoGEF Vav2⇧	Zhu et al. (2015)
Amphetamine		Cells	SK-N-SH cells	RhoA ⇧ ROCK⇧	Wheeler et al. (2015)
		Swiss-Webster mice	Midbrain slices	RhoA ⇧ ROCK⇧	Wheeler et al. (2015)
Methamphetamine	Rearing and sniffing	Sprague–Dawley rats	Nucleus accumbens	ROCK⇧	Narita et al. (2003)
	Conditioned place preference	C57BL/6J mice	Nucleus accumbens	Rac1⇧ PAK1⇧ and Cdc42⇩	Tu et al. (2019) and Zhao et al. (2019)
	Neurotoxicity	Sprague Dawley rats	Hippocampus	RhoA⇧, ROCK⇧, cofilin⇧, p-cofilin⇧	Xue et al. (2019)
Nicotine	Neurotoxicity	Cells	PC 12 cells	RhoA⇧, ROCK⇧	Fukuda et al. (2005)
Alcohol	Alcohol preference	Wistar rats	Striatum	ROCK⇩	Kurt et al. (2015)
	Alcohol preference	Wistar rats	Hippocampus	ROCK⇧	Kurt et al. (2015)
	Self-administration	Drosophila		Rac1⇩	Butts et al. (2019)
	Sedation	Drosophila		RhoGAP18b⇧ Rac1⇩ RhoA⇩ Cdc42⇧	Rothenfluh et al. (2006)
	Self-administration	Drosophila		Arf6⇧ Rac1⇧	Peru et al. (2012)

### 4.1. Rho GTPases and mesolimbic dopamine (DA) system

Dopamine (DA) has long been considered as an important regulatory neurotransmitter, regulating salience encoding, memory expression, reward prediction, and addiction (Langdon and Daw, 2020; Lin et al., 2020, 2021). Both reward and aversion stimuli activate dopaminergic neurons and promote the release of DA (Lin et al., 2020; Liu et al., 2022). The mesolimbic DA system, the dopaminergic neurons in the ventral tegmental area (VTA) project to the brain regions involved in emotional, motivational, and executive functions such as the nucleus accumbens (NAc), amygdale, and prefrontal cortex (PFC) (Serafini et al., 2020). In the field of substance dependence, the mesolimbic DAsystem is the common pathway for addictive substances to induce reward effect, and it is involved in the positive reinforcement effect, pathological memory, craving caused by addictive substances, and emotional reactions such as anxiety and fear after withdrawal (Natividad et al., 2012). Addictive substances can directly or indirectly excite VTA and cause a rapid and extensive DA release, thereby producing a rewarding effect (di Volo et al., 2019; Christoffel et al., 2021). Repeated DA treatments, which could mimic the increase of DA in synaptic transmission, increased the dendritic branching spine density of primary cortex neurons (Li J. et al., 2015). The application of DA can significantly activate Rac1 whereas decrease RhoA activity (Li J. et al., 2015). At the same time, SCH23390, the inhibitor of dopamine receptor 1 (DRD1), could inhibit the morphogenesis of dendrites and spines induced by DA treatment, and SKF81297, an agonist of DRD1, could promote neuronal morphogenesis (Li J. et al., 2015). Further results have shown that the activation of Rac1 had a positive effect on DA-induced morphogenesis, while the activation of RhoA has a negative effect, and Rac1 could interact with RhoA and then inhibit RhoA activity (Li J. et al., 2015), indicating the activation of Rac1 was a necessary condition for DA to induce RhoA inactivation, and Rac1 and RhoA had different roles in the regulation of dendritic morphogenesis following DA stimulation.

### 4.2. Rho GTPases in morphine dependence

**Figure 4:**
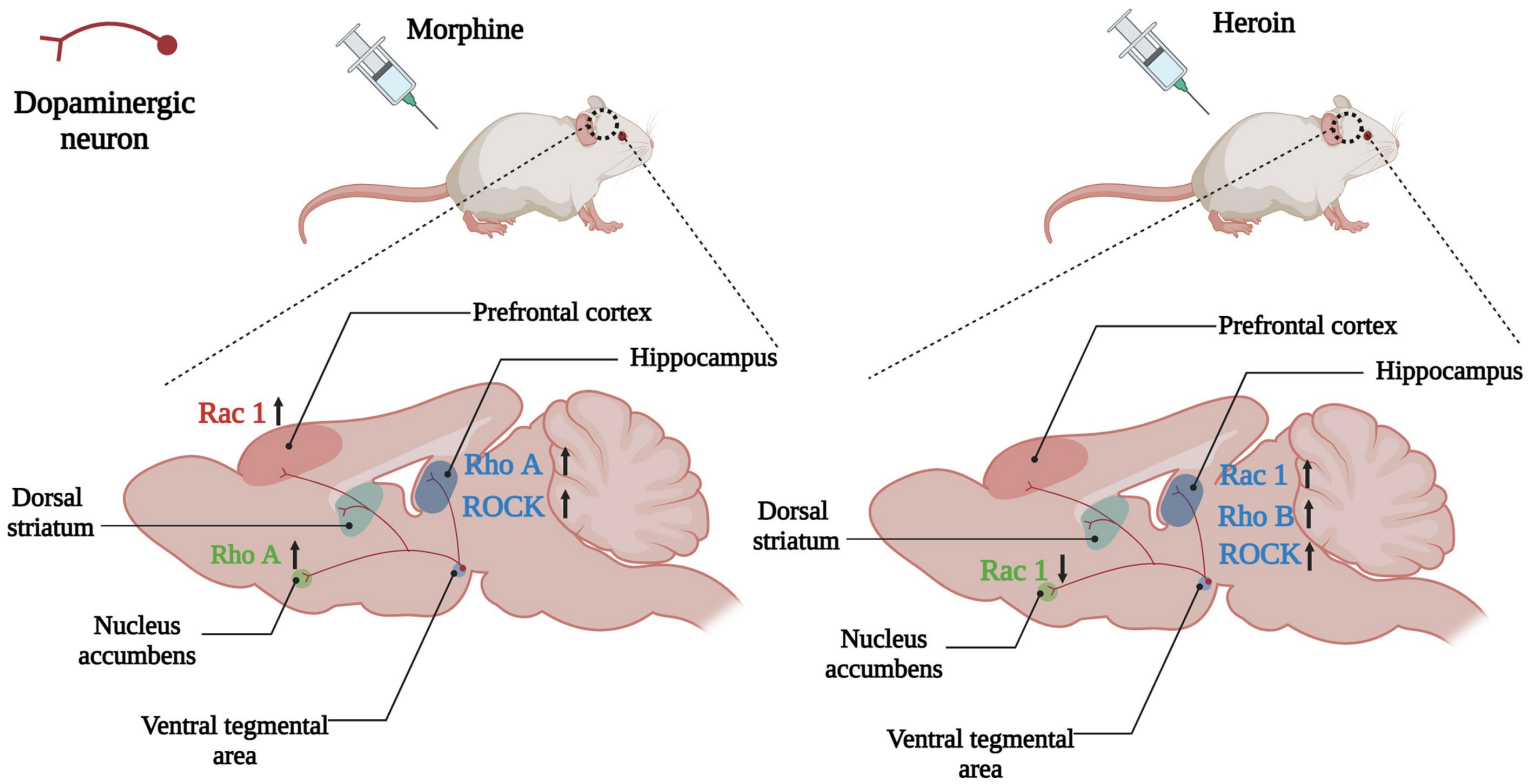
Changes of Rho GTPases expression in brain induced by opioids. In the mesolimbic dopamine system, the dopaminergic neurons in the ventral tegmental area (VTA) project to the brain regions involved in emotional, motivational, and executive functions such as the nucleus accumbens, hippocampus, dorsal striatum and prefrontal cortex. Opioids such as morphine and heroin abuse lead to changes in expressions of Rho GTPases in the mesencephalic limbic dopamine system, which in turn cause cytoskeleton remodeling and synaptic morphological and functional changes of dopamine neurons, leading to addiction and relapse. Arrows are used to represent changes in Rho GTPases and their downstream molecules in different brain regions caused by addictive substances, with upward arrows indicating increased expression and downward arrows indicating decreased expression. The color of the Rho GTPases and their downstream molecules coincides with the color of the brain regions in which they are differentially expressed. For example, the pink Rac1 in the left panel indicates changes in Rac1 expression in the prefrontal cortex (also the pink brain region) caused by morphine, and the upward arrow in the back indicates increased Rac1 expression in the prefrontal cortex caused by morphine.

Growing evidence indicated that Rho GTPases were intracellular targets of addictive substances and were involved in drug-induced dendritic spines morphogenic changes and behavior changes. After morphine conditional place preference (CPP) training, the number of hippocampal thin dendritic spines was significantly reduced, and the expression of RhoA on synapses increased, and morphine-induced CPP could be prevented by microinjection of the Rho-associated protein kinase 1 (ROCK) inhibitor H1152 to block RhoA signaling (Fakira et al., 2016), showing that the RhoA/ROCK signaling pathway was involved in the formation of morphine-induced place preference, and local inhibition of this pathway in the hippocampus completely prevents morphine-associated environmental cues (Figure 4). Although acute morphine withdrawal did not alter the synaptoneurosomal expression of the RhoA pathway, the increased expression of the RhoA network in NAc has excessively engaged and facilitated the selective elimination of thin spines on medium-sized spiny neurons (MSNs) after 2 weeks of morphine withdrawal (Cahill et al., 2018), suggesting heightened RhoA signaling pathway had a potential contribution to the elimination of thin spines during the protracted stages of morphine withdrawal.

PFC receives dopaminergic projection from VTA and is an important brain region in the reward circuit of drug addiction. The excitability of neurons in PFC is affected by the amplitude of action potential afterhyperpolarization, suggesting that they may be regulated by small conductance calcium activated potassium (SK) channels (Shan et al., 2019), which may influence somatic excitability by promoting afterhyperpolarization and regulating synaptic plasticity. It has been shown that the excitability of the fifth layer of pyramidal neurons in infralimbic (IL) regions of the PFC was decreased at 1 week after withdrawal from morphine CPP training, and the expression of the SK3 and activated-Rac1 were enhanced compared to controls (Qu et al., 2020). Meanwhile, NSC23766, one of Rac1 inhibitors, could disrupt SK current and increase neuronal firing, while downregulation of Rac1 reduced the expression of SK channels in IL and inhibited morphine-induced CPP (Qu et al., 2020), indicating that Rac1 signaling pathway reduced neuronal excitability by regulating SK channel in PFC after morphine withdrawal, and this could help shed light on the mechanisms of opioid dependence and the development of treatment strategies.

Although the current neurobiological mechanism of opioid abuse is primarily related to synaptic plasticity of dopaminergic and glutaminergic neurons, this drug-induced plasticity may also be enhanced by glial cell activity. More and more evidence showed that glial cells, including oligodendrocytes, astrocytes and microglia, participate in cognitive impairment-and drug-induced neuronal plasticity changes, through the activity of cytokines and neurotrophins (Rahman et al., 2022). Chronic morphine therapy induces activation of astrocytes and microglia (Valentinova et al., 2019). In addition to affecting neuronal plasticity, Rac activity in glial cells is also involved in morphine addiction. Repeated morphine exposure could induce the activation of astrocytes and microglia *in vivo*, and promote the release of cytokines and glutamate from neurons. At the same time, morphine could induce morphological changes of cultured microglia from bipolar rod-like shapes or globular to lamellipodial and flat, with membrane ruffling at the edge, which was colocalized with Rac (Takayama and Ueda, 2005). These results indicated that Rac activation was involved in morphine-induced changes in neuronal synaptic plasticity and glial cell activation, which all affected morphine induced abnormal behaviors.

### 4.3. Rho GTPases in heroin dependence

A key point with substance dependence is that abuse usually occurs in specific situations, therefore, exposure to the environment associated with addictive substance can increase craving and the odds of relapse, even after long-term withdrawal (Hitchcock and Lattal, 2018). Many brain regions play a role in environmental context-induced reinstatement of drug seeking and relapse, including dorsal and ventral PFC, dorsal striatum, basolateral amygdala and hippocampus (Marchant et al., 2015; Pelloux et al., 2018; Sun and Giocomo, 2022). Among them Hippocampus is crucial for developing and retrieving contextual and spatial memories (Sun and Giocomo, 2022). The dorsal hippocampus (DH) is the physiological basis for the learning of associations between the environmental context and unconditioned stimuli (such as drug use), and it is involved in mediating drug-taking and drug-seeking behavior and relapse (Chen et al., 2017; Hitchcock and Lattal, 2018). The expression of RhoB was increased in the DH of heroin self-administering rats, whereas blocking the RhoB/ROCK network could attenuate context-induced heroin relapse (Chen et al., 2017; Figure 4), which indicated that the RhoB pathway in the DH was necessary for the retrieval (recall) of addiction memory.

Rac1 was also particularly needed in cytoskeleton remodeling of the dendritic spine. DNA methylation is an essential mechanism for controlling gene expression, and it is essential for long-term synaptic plasticity. Calcium/calmodulin-dependent kinases (CaMKs) have become fundamental molecules that control spinal morphogenesis and dendritic development (Chang et al., 2019; Wang et al., 2019; Shibata et al., 2021). CaMKII is critical to the plasticity of activity-dependent spines (Yasuda et al., 2022). In addition to CaMKII, neurons also contain other CaMK cascades, facilitated by CaMK kinase (CaMKK) and its major downstream targets. Self-administration of heroin increased the expression of CaMKK1, which in turn activated its downstream target CaMKIα. The CaMKK1/CaMKIα pathway activated Rac1 *via* interacting with βPIX (Rac GEF) to regulate actin cytoskeleton remodeling in the DH and behavioral plasticity (Chen et al., 2021). Repeated self-administration of heroin caused the activation of Rac1 and the ratio of F-actin to G-actin in the DH increased significantly, indicating that the actin cytoskeleton was remodeled (actin polymerization). More importantly, bilateral intra-DH injection of NSC23766 significantly blocked actin polymerization and inhibited heroin self-administration (Chen et al., 2021), demonstrating that the upregulation of CaMKK1 in the DH was related to the motivation of heroin intake, and CaMKK1/CaMKIα/Rac1 pathway was required for the cytoskeleton remodeling and heroin addiction behavior of rats. However, Autism susceptibility candidate 2 (AUTS2) could inhibit the initiation and expression of heroin-induced behavioral sensitization *via* elevating Rac1 activation in NAc (Zhu et al., 2016). These results demonstrate that actin cytoskeleton remodeling is necessary for heroin addiction and hyperactive behavior, though the Rac1 signaling pathway may play different roles in different regions.

### 4.4. Rho GTPases in cocaine dependence

“Goal-directed” actions, the ability to choose actions based on expected consequences, are critical for the survival of individuals, which means that their performance is sensitive to the change in the connections between the action and its results. Habit-based stimulus-induced reward seeking at the expense of “goal-directed” response strategies may play an etiological role in the development and maintenance of substance dependence, especially for cocaine addiction (Everitt and Robbins, 2016). The cortex-dorsomedial striatum circuit is critical to the acquisition of “goal-directed” actions (Hart et al., 2014), and lesions of the prelimbic cortex impaired the ability of laboratory animals to choose actions based on previously encoded action-outcome associations (Corbit and Balleine, 2003), indicating the prelimbic prefrontal cortex is essential for making “goal-oriented” decisions. Swanson et al. found that decision-making strategies could be predicted by dendrite spine density, and the intensity of action-outcome conditioning correlated with dendrite spine density in the prelimbic cortex, suggesting that the structural plasticity of prelimbic cortical neurons may play critical roles in the generation of “goal-directed” actions (Swanson et al., 2017). Systemic and local prelimbic cortical injection of the fasudil (ROCK inhibitor) could block the habitual response to food and cocaine in an actin polymerization-dependent manner, and temporarily reduce dendritic spine densities in prelimbic cortex in the process of consolidating new action-outcome associations (Swanson et al., 2017), demonstrating that blocking Rho/ROCK could impact the habit-based behaviors, including in the context of cocaine habits. HA-1077 (ROCK inhibitor) treatment during the adolescent period exaggerated cocaine-induced locomotor activity in adulthood, and administration of HA-1077 in adulthood had no psychomotor consequences in mice (DePoy et al., 2013), suggesting that early-life ROCK inhibition for destabilizing dendritic spines could confer cocaine vulnerability.

**Figure 5:**
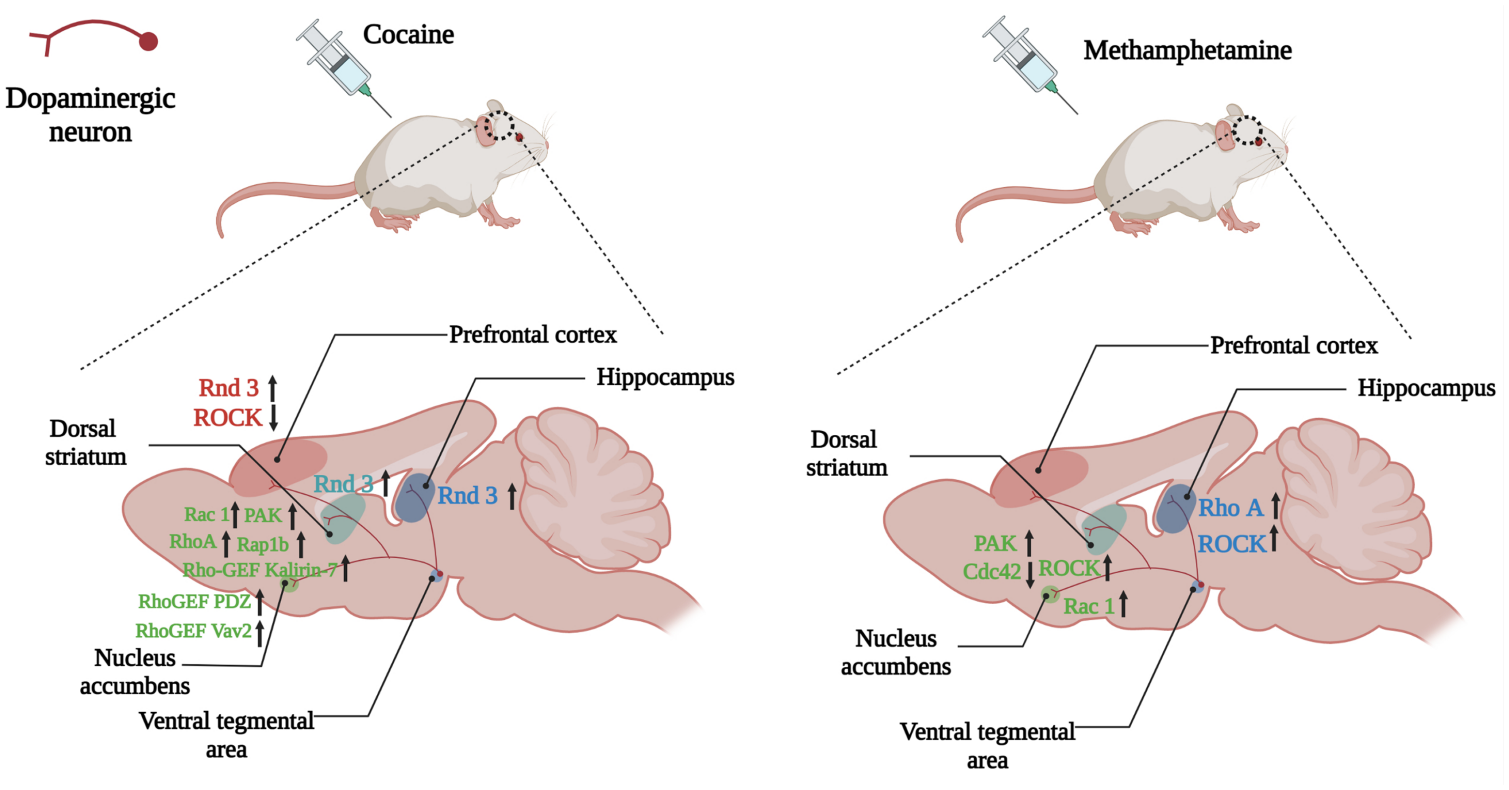
Changes of Rho GTPases expression in brain induced by psychostimulants. Dopamine plays a role in the brain’s reward system, helping to reinforce certain behaviors that result in reward. Dopaminergic neuronal projections from the ventral tegmental area (VTA) to the nucleus accumbens, dorsal striatum and hippocampus, and prefrontal cortex underlie the neurobiology of this process. Psychostimulants such as cocaine and amphetamine lead to abnormal expressions of Rho GTPases (Rho A, Rac1, Rnd3) and their regulators (Kalirin-7, Vav2) in the above regions, which take part in the reinforcement, withdrawal and relapse of psychostimulants addiction. Arrows are used to represent changes in Rho GTPases and their regulators in different brain regions caused by addictive substances, with upward arrows indicating increased expression and downward arrows indicating decreased expression. The color of the Rho GTPases and their downstream molecules coincides with the color of the brain regions in which they are differentially expressed.

Cocaine addiction leads to persistent alterations in the brain’s reward system, including increased dendrites and spine density on MSNs in the NAc and glutamatergic projection neurons in the PFC and ventral hippocampus (Li et al., 2012; Barrientos et al., 2018). Except Rho/ROCK pathway, the members of the Rnd subfamily also play essential roles in the regulation of actin cytoskeleton. Different from other small Rho GTPases, members of the Rnd subfamily (Rnd1, Rnd2, and Rnd3) do not hydrolyze GTP, therefore, Rnd proteins are not regulated by GAPs, and might be mainly regulated by transcriptional modulation (Chardin, 2003). Rnd3 is ubiquitously expressed, while Rnd1 and Rnd2 are mainly expressed in the brain. Studies have shown that Rnd1 promoted the maturation of the dendrite spine, Rnd2 stimulated dendrite branching, and Rnd3 regulated cytoskeleton remodeling and cell migration. The level of Rnd3 mRNA in the dorsal striatum, PFC and, hippocampus was significantly increased after the cocaine injection (Marie-Claire et al., 2007; Figure 5). Considering the role of the Rnd subfamily in actin cytoskeleton modulation and synaptic plasticity, cocaine regulation of Rnd3 gene expression suggests that Rnd3 is involved in the growth of neurites and modification of dendritic branches induced by cocaine abuse (Marie-Claire et al., 2007). With limited research progress in this field, it would be significant to study the direct relationship between Rnd3 and cocaine addiction and neuronal morphologic changes.

As positive regulators of Rho GTPases, Rho GEFs also become central regulators of synaptic actin dynamics. Kalirins, a well-known subfamily of Rho-GEFs promote the exchange of GDP and GTP, thereby stimulating the activity of specific Rho GTPases (Paskus et al., 2020). Kalirin-7 (Kal-7) is located on the postsynaptic side of excitatory synapses, and it is the most abundant Kalirin subtype in adult rodent brains. Kal-7 could activate Rac1, RhoA, and RhoG and is critical to the formation and stability of dendritic spines (Ma, 2010; Mazzone et al., 2012). The binding of the PH domain of Kal-7 and the NR2B (one of glutamate receptor NMDAR subunits) was necessary for synaptic plasticity (Kiraly et al., 2011). Chronic cocaine induced an increased expression of Kal-7 in the NAc of wild-type mice, and overexpression of Kal-7 in cultured MSNs increased the density of dendritic spine, while knocking out of Kal-7 inhibited the increase in spine density after cocaine treatment, indicating that Kal-7 was supposed to be indispensable for increases of dendritic spine density induced by cocaine (Kiraly et al., 2010; Ma et al., 2012). Lack of Kal-7 did not influence acute locomotor response to cocaine, but it could increase cocaine locomotor sensitization and self-administration (Kiraly et al., 2010, 2013; LaRese et al., 2017). After 2 weeks withdrawal from repeated cocaine injections, the levels Kal-7 and the activation of its downstream effectors such as Rac1 and PAK in the NAc were increased, while the surface expression of glutamate receptor AMPAR and spine density also increased (Wang et al., 2013). Kal-7 knockdown eliminated the increased expression of AMPAR and spine density, and Kal-7 knockdown rats also exhibited locomotor sensitization, however, incentive sensitization, which was assessed by the speed rats learned to self-administer a threshold dose of cocaine, was impaired severely (Wang et al., 2013). Blockade of NR2B with ifenprodil also inhibited locomotor hypersensitization of cocaine in Kal-7^KO^ mice (LaRese et al., 2017). These results indicated that Kal-7/Rac1/PAK pathway coordinated glutamate receptors to increase dendritic spine density during cocaine withdrawal, while incentive sensitization and locomotor sensitization may involve divergent mechanisms.

The synaptic structure and function of NAc MSNs in the early and late stages during cocaine withdrawal had opposing alterations. For instance, the observation of *de novo* thin spine formation at early time points indicated weakened synapses, while the increased formation of mushroom dendritic spines and related strengthened synapses were seen at later time points. After long-term exposure to cocaine, the level of PDZ-RhoGEF (one of the RhoGEFs, encoded by Arhgef11 gene) in the cytoplasm of NAc MSNs decreased, and the level of PDZ-RhoGEF in the nucleus increased (Cahill et al., 2016). This change was related to the increase in the levels of active RhoA in the nucleus, which in turn promoted the formation of F-actin. Meanwhile, the mRNA level and the synaptoneurosomal fraction level of Rap1b, which belongs to the Rap subfamily, were selectively increased after cocaine exposure, and further studies demonstrated that the elevated levels of active RhoA and Rap1b were related to the upregulation of PDZ-RhoGEF (Cahill et al., 2016). NAc infusion of Rap1b inhibitor GGTI-298 reduced locomotor activity and CPP induced by cocaine, and Rap1b could activate the PI3K-Akt-mTOR pathway, which, in turn, promoted the formation of thin spines at early time points of cocaine withdrawal. On the contrary, the activity of this pathway was reversed at late time points of cocaine withdrawal, resulting in the formation of mushroom spines (Cahill et al., 2016). These findings reveal the key role of biaxial synaptic expression of PDZ-RhoGEF/Rap1b in cocaine addiction. Although the actin dynamic changes that occur early and late time points appear to be different, synaptic remodeling during this process is a potential example of “superplasticity,” with the initial formation of new fine spines early providing sites where subsequent dendritic spine maturation occurs (Wright et al., 2020).

Dopamine transporter (DAT)-mediated DA reuptake is one of the most critical mechanisms for substance dependence. Vav2 belongs to the VAV family with guanine nucleotide exchange activity. Compared with wild-type mice, the surface expression of DAT in the NAc of cocaine-treated Vav2^−/^- mice was significantly reduced, along with reduced DA levels and diminished behavioral response to cocaine (Zhu et al., 2015), demonstrating that Vav2 was a key factor of DAT trafficking and cocaine abuse.

### 4.5. Rho GTPases in amphetamine dependence

Amphetamine is a psychostimulant that can increase DA content in the synaptic cleft by inhibiting DA reuptake in dopaminergic neuron terminals, and DAT is the crucial target of amphetamine actions during addiction. Different from cocaine and other psychostimulants, amphetamine can serve as a DAT substrate to stimulate the endocytosis of DAT in the plasma membrane. For instance, after injections of amphetamine (2 mg/kg), the level of DAT on the cell membrane of the midbrain slice significantly decreased (Saunders and Galli, 2015; Wheeler et al., 2015), which may be the key mechanism that leads to addiction. Wheeler et al. demonstrated that amphetamine rapidly stimulated DAT trafficking through a clathrin-independent, dynamin-dependent process in midbrain slices and cultured dopamine neurons, and this effect was mediated by activation of the RhoA (Wheeler et al., 2015). Inhibiting RhoA activity by C3 exotoxin, ROCK inhibitor Y27632, or a dominant-negative RhoA could block amphetamine-induced DAT internalization and dopamine uptake. Amphetamine also stimulated the accumulation of cAMP and PKA-dependent inactivation of RhoA, thus regulating the timing and stability of the effect of amphetamine on DAT internalization through the interaction of PKA and RhoA signaling pathways (Wheeler et al., 2015). The regulation of Rho activation/inactivation sequence provided a mechanism through which endogenous neurotransmitters and drugs could affect the response of dopaminergic neurons to amphetamine (Figure 5).

Similar to DAT, which is responsible for maintaining the DA concentration in the synaptic cleft, excitatory amino acid transporters (EAATs) are responsible for regulating the concentration of extracellular glutamate, which determines the spatial and temporal precision of glutamate neurotransmission and limits the excitotoxic effects of glutamate. EAAT3 is found predominantly in neurons and is the main subtype of glutamate transporter in dopaminergic neurons (Shin et al., 2020). Amphetamine stimulated the internalization of EAAT3, which was dependent on the activation of a RhoA, dynamin, and the expression of DAT, and decreased glutamate uptake in cultured neurons (Underhill et al., 2014). EAAT3 Trafficking from the cytomembrane after amphetamine treatment enhanced glutamate synaptic transmission by reducing glutamate clearance, proving a new mechanism for amphetamine to regulate midbrain glutamate activities.

NAc injection of ROCK inhibitor Y27632 significantly suppressed the increase of extracellular DA levels and behaviors induced by methamphetamine (METH) (Narita et al., 2003). METH regulated the Rac1 and Cdc42 signaling pathways oppositely, and inhibiting the Rac1 signaling and activating the Cdc42 signaling were crucial to CPP and structural plasticity induced by METH. DRD1 activated both the Rac1 and Cdc42 signaling, while DRD2 activated Cdc42 signaling but inhibited Rac1 signaling, thereby mediating METH-induced CPP and structural plasticity (Tu et al., 2019). These results showed both DRD1 and DRD2 in the MSNs of NAc regulated METH-induced locomotor activation, CPP, and dendritic and spine remodeling, however the possible mechanisms may differ.

The acquisition of METH-related addiction memory increased the thin dendritic spine density of DRD1-and DRD2-MSNs and it decreased the activity of Rac1 and the expressions of its downstream p-PAK, p-Cofilin, and p-LIMK, whereas overexpression of Rac1 accelerated the extinction of METH-associated contextual memory. Additionally, although NAc microinjection of Rac1 inhibitor or activator was not sufficient to interrupt the reconsolidation of METH-associated contextual memory, the pharmacological activation of Rac1 in the NAc also promoted the elimination of thin spines and the extinction of METH-associated contextual memory. It was worth noting that Rac1 had an effect on the spine plasticity of DRD1-MSNs induced by METH, but had no effect on DRD2-MSNs. These findings indicated that Rac1 might play an opposite role in the acquisition and extinction of METH-related contextual memory, and the role of Rac1 in METH-related spine remodeling is also cell-specific, indicating that Rac1 may be a potential therapeutic target for reducing METH relapse (Zhao et al., 2019).

In addition to addiction and hyperactivity, METH is highly neurotoxic to the central nervous system. METH significantly increased blood-brain barrier (BBB) permeability, induced cytoskeleton remodeling, and reduced levels of tight junction (TJ) proteins in the hippocampus. The expressions of RhoA, ROCK, myosin light chain (MLC), p-MLC, cofilin, p-cofilin, and matrix metalloproteinase (MMP)-9 were all up-regulated, indicating that the RhoA/ROCK pathway was activated after METH treatment. After pretreatments with RhoA or ROCK inhibitors, the expressions of above-mentioned proteins significantly decreased, and the expressions of TJ proteins were increased, while the rearrangement F-actin cytoskeleton was suppressed and the permeability of rat brain microvascular endothelial cells was also reduced. These results indicated that METH could induce cytoskeleton rearrangement and TJ protein down-regulation *via* activating the RhoA/ROCK pathway, and then increase the permeability of BBB (Xue et al., 2019).

The neurotoxicity caused by METH abuse is associated with neurodegenerative damage in various brain regions such as the hippocampus and dorsal striatum. For instance, METH can cause dopaminergic neurons apoptosis, and using small interfering RNA to silence ROCK2 could increase cell viabilities and reduce apoptosis of PC12 cells (Yang et al., 2013), showing that ROCK2 may be a possible target for the treatment of METH-induced neurotoxicity. METH induced cytomorphological changes on human neuroblastoma SH-SY5Y cells including macropinocytosis through actin-dependent endocytosis (Nara et al., 2010). Further study showed that macropinosomes formed after METH exposure colocalized with activated Ras and activated Rac1, and both Rac1 inhibitor and Ras inhibitor significantly suppressed the formation of macropinosomes. Meanwhile, the levels of lysosomal-associated membrane proteins increased gradually in a time-dependent manner after METH exposure, while the proteolytic activities of cathepsin L were significantly suppressed, indicating that lysosomal function was inhibited. These results indicate that inhibition of lysosomal function through Ras-and Rac1-mediated macropinocytosis is at least partially involved in cytotoxic effects of METH (Nara et al., 2012).

### 4.6. Rho GTPases in alcohol dependence

Alcohol has been widely abused in many cultures for centuries, and drinking alcohol is related to the risk of chronic diseases including mental and behavioral disorders, liver cirrhosis, and cardiovascular diseases. Studies have found that the activity of ROCK in the striatum of rats was significantly reduced after being exposed to alcohol, while that in the hippocampus was significantly increased (Kurt et al., 2015), indicating that alcohol had different effects on the Rho/ROCK pathway in different brain regions. Rac1 and its target cofilin could bi-directionally change the experience-dependent alcohol preference. Dominant-negative Rac1 or activated cofilin in the mushroom bodies of Drosophila led to faster acquisition of experience-dependent alcohol preference, while activated Rac1 or dominant-negative cofilin eliminated alcohol preference, suggesting the activation state of Rac1 and cofilin were key factors to determining the rate of acquisition of alcohol preference and actin dynamics regulation played critical roles in the development of voluntary self-administration in Drosophila (Butts et al., 2019).

Low doses of alcohol (15% alcohol in the preference assays; Butts et al., 2019) caused disinhibition and hyperactivity (increased locomotion), while higher doses (alcohol vapors; Rothenfluh et al., 2006) led to dyskinesia and subsequent sedation. Small GTPase Arf6 acts downstream of Rac1 and Arfaptin and functions in the nervous system of adults. Arfaptin can directly bind to the activated Rac1 and Arf6, and Arfaptin and Arf6 mutations caused hypersensitivity to alcohol-induced sedation, demonstrating that the conservative Rac1/Arfaptin/Arf6 pathway was the main mediator of behavioral responses to alcohol in Drosophila (Peru et al., 2012).

The biological response to alcohol is highly conserved, and the reduced response to the sedative effects of alcohol indicates an increased risk of alcohol dependence in humans. RhoGAP18B is one of Rho GAPs and also affects the sensitivity to alcohol. Blockage of RhoGAP18B has strong resistance to the sedative effect of alcohol, and could enhance the GTPase activities of human Cdc42 and Rac, but has no impacts on RhoA. This resistance can be inhibited by decreasing the levels of RhoA or Rac1, indicating these GTPases were involved in the behavioral response to alcohol (Rothenfluh et al., 2006). The loss-of-function mutation in cofilin led to decreased alcohol-sensitivity and synergistic effect with RhoGAP18B, and the RhoGAP18B-PA subtype acted on Cdc42, while PC and PD activated cofilin through Rac1 and Rho, indicating that different RhoGAP18B subtypes acted on different Rho GTPases subgroups to regulate actin polymerization and depolymerization, and alcohol-induced behaviors (Ojelade et al., 2015).

The central nervous system has mechanisms to ensure the process of forming appropriate neuron polarity and regulating the growth time of the processes, and alcohol can destroy these mechanisms and alter the normal establishment of neuronal polarity. For instance, cultured pyramidal neurons of the fetal rat hippocampus developed abnormally small dendritic branches after being exposed to alcohol in the early stages of cultivation (Clamp and Lindsley, 1998), and long term alcohol treatment led to a reduction in the total length of dendrites, the number of dendrites and the number of synapses (Yanni and Lindsley, 2000). Ca^2+^ signaling pathway is a key regulator of axon growth and guidance, altered Ca^2+^ signaling in growth cone were associated with abnormal neuromorphogenesis caused by fetal alcohol exposure (Mah et al., 2011). Alcohol exposure inhibited Rac1/Cdc42 activation induced by BDNF in a dose-dependent manner, and in the early stages of hippocampus development, alcohol inhibited growth cone signaling through regulating the activities of small Rho GTPases (Lindsley et al., 2011), suggesting alcohol may destroy the effect of neurotrophic factors on axon growth and guidance. Exposure 7-day-old rat pups to alcohol inhibited the formation of neurite and activation of Rac1 in cerebellar granule neurons, demonstrating that Rho GTPases played a regulatory role in the differentiation of cerebellar neurons, and alcohol-related impairment of Rac1 signaling may be one of the causes of brain defects observed in fetal alcohol syndrome (Joshi et al., 2006).

### 4.7. Rho GTPases in nicotine dependence

Nicotine dependence is one of the most expensive health problems worldwide. A microarray study on the time-course of acute response to nicotine in the VTA of mice showed that RhoA expression levels increased significantly with the duration of nicotine treatment, and one SNP of RhoA gene, rs2878298, showed a highly significant genotypic association with the initiation of smoking and nicotine dependence (Chen et al., 2007). Given the role of RhoA in cytoskeleton rearrangement and formation of dendritic spines, these results suggested RhoA’s regulation of neuroplasticity may be involved in nicotine abuse. Tyrosine hydroxylase (TH) is a key rate-limiting enzyme in the biosynthesis of catecholamine such as DA. Nicotine could significantly activate RhoA and increase the expression and enzyme activity of TH in culture rat pheochromocytoma PC 12 cells, and treatment with Y27632 or C3 toxin significantly to block RhoA signaling remarkably inhibited the nicotine-induced increase of TH and catecholamine biosynthesis in PC12 cells (Fukuda et al., 2005), indicating RhoA may be involved in the increase of catecholamine neurotransmitters such as dopamine caused by nicotine and the activation of reward circuits.

## 5. Considerations for clinical use and outlook

The Rho/ROCK pathway is abnormally activated in various diseases of the central nervous system, and blocking of the Rho/ROCK pathway has been shown to be effective in animal models of Alzheimer’s disease, neuropathic pain and stroke (Mueller et al., 2005). ROCK was also abnormally expressed in many brain regions of the addicted animals, therefore, inhibitors of ROCK may also have the potential to treat drug abuse. To date, only two inhibitors of ROCK, fasudil (Eril^®^) and its derivative ripasudil (Glanatec^®^), have been approved for clinical use. Fasudil is a small molecule ROCK inhibitor, which was first approved in 1995 in Japan for the prevention and treatment of cerebral vasospasm and subarachnoid hemorrhage (Abedi et al., 2020). Fasudil has been proved to stimulate neuroregeneration and prevent neurodegeneration in many neurological diseases (Tatenhorst et al., 2016). Fasudil could enhance action-outcome memory, leading to “goal-directed” behavior in mice, otherwise the mice would express “stimulus-response” habits, and fasudil can also prevent the habitual response to cocaine, and this effect would persist over a period of time based on the polymerized state of actin (Swanson et al., 2017). Fasudil administration could significantly ameliorate spatial learning and memory disorders and reduce the increase of inflammatory cytokines levels induced by smoking in the hippocampus, which may be related to the regulation of the Rho/ROCK/NF-κB pathway (Xueyang et al., 2016). In 2014, Pharmaceuticals and Medical DevicesAgency of Japan approved Ripasudil as an ophthalmic solution for the treatment of glaucoma with increased intraocular pressure (Koch et al., 2018). However, there have been no reports of ripasudil and drug addiction. RhoA activation may be involved in the up-regulating of the expression and activity of ileal P-gp protein, resulting in a decrease in the analgesic effect of oral morphine (Kobori et al., 2012), and inhibition of RhoA activation by rosuvastatin not only delayed, but also partially reversed the morphine tolerance (Li Y. et al., 2015).

Substance dependence is associated with mental disorders such as depression and anxiety, which are major factors leading to compulsive drug seeking and relapse. Opipramol, a sigma-1 receptor agonist, is approved in some European countries to treat anxiety disorders and depression. It has been reported that chronic opipramol administration could reduce cocain-seeking behavior in self- self-administrated rats. Further results suggested that Rac1 played a key role in the effect of opipramol on drug seeking, affecting the susceptibility of addicted rats to opipramol (Bareli et al., 2021b). As a drug used in clinical practice, opipramol reduced Rac1 and inhibited cocaine relapse, indicating its potential as a candidate for the treatment of substance dependence. Opipramol combined with baclofen significantly reduced cravings and depressive symptoms in substance abusers, according to a preliminary, double-blind, controlled clinical investigation. These findings suggest that opipramol and baclofen could be candidates for treatment in patients with substance abuse and comorbidities for mood/anxiety disorders, which may help improve their addiction status and promote rehabilitation (Bareli et al., 2021a).

Although there are preclinical studies that support the beneficial effects of Rac1, RhoA/ROCK inhibitors on drug abuse, human clinical studies and data confirming their beneficial effects are limited. More clinical trials are needed to determine whether fasudil or opipramol show beneficial effects in the treatment of drug dependence clinically and to research and develop more effective Rho GTPase- related compounds.

## 6. Conclusion

Accumulating studies have indicated that the Rho GTPases family and their regulatory factors regulators, in particular GEFs and GAPs, play crucial roles in substance dependence, which provide novel insights into the treatment. However, our work cannot tell the whole story because more and more findings are being discovered about the role of Rho GTPases and their regulators and effects in substance dependence, and even more have yet to be revealed. The cell specificity and brain region specificity of Rho GTPases indicate we remain far from a complete understanding of how they operate spatially and temporally in neuronal structure and function in substance dependence. A better understanding of regulatory mechanisms of these Rho GTPases will certainly highlight novel therapeutic targets and interventions for the treatment of substance dependence.

## Author contributions

QR, LC, and YXW designed the study and wrote the manuscript. All authors contributed to the article and approved the final version.

## Funding

This work was supported by the National Natural Science Foundation of China (Grant No. 81971775) to LC and (Grant No. 82071970) to YXW, the Young Talents Project of Hubei Provincial Health Commission (Grant No. WJ2021Q053) to QR, and the Science and Technology Project of Jianghan University (Grant No. 2022SXZX25).

## Conflict of interest

The authors declare that the research was conducted in the absence of any commercial or financial relationships that could be construed as a potential conflict of interest.

## Publisher’s note

All claims expressed in this article are solely those of the authors and do not necessarily represent those of their affiliated organizations, or those of the publisher, the editors and the reviewers. Any product that may be evaluated in this article, or claim that may be made by its manufacturer, is not guaranteed or endorsed by the publisher.
